# In COM we trust: Feasibility of USB-based event marking

**DOI:** 10.3758/s13428-021-01571-z

**Published:** 2021-04-14

**Authors:** Stefan Appelhoff, Tristan Stenner

**Affiliations:** 1 Center for Adaptive Rationality, Max Planck Institute for Human Development, Lentzeallee 94, 14195 Berlin, Germany; 2 Institute of Medical Psychology and Medical Sociology, University Medical Center Schleswig Holstein, Kiel University, Kiel, Germany

**Keywords:** Event marking, Parallel port, USB, TTL, Trigger, Open-hardware

## Abstract

Modern experimental research often relies on the synchronization of different events prior to data analysis. One way of achieving synchronization involves marking distinct events with electrical pulses (event markers or “TTL pulses”), which are continuously recorded with research hardware, and can later be temporally aligned. Traditionally, this event marking was often performed using the parallel port in standard personal computers. However, the parallel port is disappearing from the landscape of computer hardware, being replaced by a serial (COM) port, namely the USB port. To find an adequate replacement for the parallel port, we evaluated four microcontroller units (MCUs) and the LabJack U3, an often-used USB data acquisition device, in terms of their latency and jitter for sending event markers in a simulated experiment on both Windows and Linux. Our results show that all four MCUs were comparable to the parallel port in terms of both latency and jitter, and consistently achieved latencies under 1 ms. With some caveats, the LabJack U3 can also achieve comparable latencies. In addition to the collected data, we share extensive documentation on how to build and use MCUs for event marking, including code examples. MCUs are a cost-effective, flexible, and performant replacement for the disappearing parallel port, enabling event marking and synchronization of data streams.

## Introduction

Experimental research in a multitude of scientific disciplines involves the presentation of stimuli to research subjects. Be it displaying a visual stimulus to a human, applying an electric shock to a rodent, or switching off the lights in a room being navigated by an autonomous robot – researchers need to record these events together with other data to make sense of the results. A traditional implementation for event marking is the parallel port available on standard computers (Clerc et al., 2016). The parallel port works with direct current signals that directly translate to digital states on the receiving interface, and can thus achieve microsecond resolution of data transmission (Stewart, 2006; Voss et al., 2007). These fast direct current signals, also called transistor–transistor logic (TTL) pulses, persuaded manufacturers of recording hardware for experimental research to adopt the parallel port as a standard interface for implementing event marking. However, the parallel port’s original role as a general interface to transmit data has been taken over by serial communication protocols, specifically the ubiquitous “universal serial bus” (USB) protocol. As parallel port interfaces are replaced by USB ports on commercially available computers, it is becoming increasingly difficult to obtain modern computer hardware that supports a parallel port “out of the box”. Yet, most data recording hardware for experimental research still relies on event marker inputs sent from a parallel port interface. Consequently, researchers often find themselves hoarding outdated computer equipment or relying on workarounds such as PCI adapter cards or old docking stations for modern laptops. The problem of parallel port availability is even more pronounced for users of Apple computers, which are traditionally produced without a parallel port interface (Knight, 1997). But even for users of recent versions of Microsoft Windows, access to the parallel port has become increasingly difficult for native applications and impossible for web browser-based studies (see e.g., Bridges et al., 2020).

Workarounds for event marking should be treated with caution: because they tend not to be exhaustively tested, they can introduce dangerous uncertainty about the true latency between an event and its associated marker in the data. Some analysis techniques such as event-related potentials (ERPs, Luck, 2014) can tolerate a small *constant* latency of a few milliseconds, but quickly become unusable with unpredictable variations of the latencies (i.e., jitter). Commercial manufacturers are beginning to acknowledge this situation and to release products tailored to bridging the link from USB connectors to the proprietary connectors expecting TTL pulse inputs as from a parallel port. Currently, there are at least seven products to choose from (e.g., Canto et al., 2011; see Supplemental Material for a list), most of which have been tested by their respective manufacturers. Unfortunately, however, most of the products have at least one of the following drawbacks: (i) they are expensive, (ii) they are specific to a particular type of hardware, or (iii) they provide supported functionality only under a limited set of operating system or software packages (i.e., hardware drivers or accompanying software are not provided for all major operating systems). Although the relatively widespread MCU evaluation boards such as Arduinos can be used for the same purpose, many labs shy away from them because of uncertainty as to whether or not events can be marked with the required precision. The same holds for commercial USB input/output products for which no published timing test results are available (Wimmer et al., 2019).

With the present study, we aim to resolve this uncertainty and to demonstrate that USB-based “event trigger devices” that present themselves as serial (COM) ports can generally serve as adequate replacements for the previous gold standard parallel port. We describe the general principle underlying the commercial products that link the USB port to proprietary connectors expecting a TTL pulse. Furthermore, we run a suite of latency tests on several such event trigger devices under two major operating systems (Windows, Linux) and compare the results against the performance of a traditional parallel port. We did not run tests under the macOS operating system because devices running macOS do typically not have native parallel port support. In the Supplemental Material, we provide a detailed tutorial on how to build event-trigger devices and software examples on how to operate them.

## General principle underlying MCUs

A microcontroller unit (MCU) consists of at least one processor, memory (both volatile working memory and programmable memory for program code), and digital in- and output ports (e.g., for TTL signals or standardized buses like USB or the Serial Peripheral Interface, SPI). Upon startup, the MCU reads the firmware from the internal memory and executes the contained instructions. The event-trigger devices analyzed here were programmed to read a trigger value from the PC, either directly via an embedded USB controller or a separate USB controller chip attached via an internal serial port, activate the corresponding output ports for a few milliseconds, and repeat these instructions from the beginning. The pseudocode for the simplest possible trigger device firmware consists of just a few lines (see Algorithm 1). This design involves at least two send- or receive-buffers (four in the case of a separate USB controller) which, combined with a fixed transfer rate and buffer sizes, put upper bounds on bandwidth (typically 14.4 kB/s at 115200 baud; 1.2 kB/s at 9600 baud) and lower bounds on latency (69.4 μs at 115200 baud with a single pair of buffers that can be flushed instantaneously up to 107 ms for two 64-byte buffers at 9600 baud). The simplest communication scheme translates one input byte to 8 bits, each of which is linked to one digital output and encoded in base 2 (e.g., the trigger 75 would translate to 0*128+1*64+0*32+0*16+1*8+0*4+1*2+1*1=01001011_2_, so the pins 7, 4, 2, and 1 would be enabled).

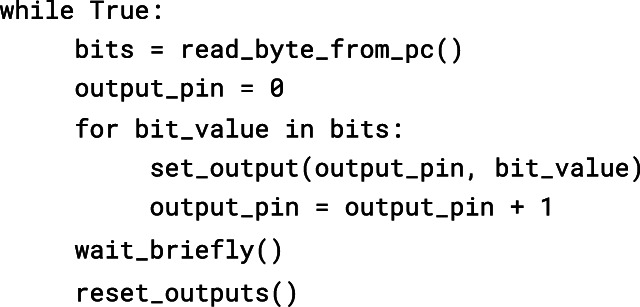



Algorithm 1. *Example pseudocode for operating an event trigger device*. Note that unlike in this example, some MCUs may have specialized functions that allow setting all output pins at once, without having to iterate through a loop.

Following the code in Algorithm 1, an event trigger (in form of a TTL pulse) can be caused by sending a single byte, the trigger value, to the device’s input buffer. More functionality can be built in by replacing the MCU’s firmware, implementing an instruction scheme that evaluates the input and sets outputs depending on the result of the computation. With minor adjustments, some MCUs can even be accessed via WebUSB to synchronize browser-based experiments with measurement devices via TTL pulses. Being able to program an MCU’s firmware to execute almost arbitrary logic and communication protocols can thus be a big advantage. However, such firmware modifications are typically not possible for commercial devices, as these are often constrained by built in, proprietary firmware. This constitutes the main difference between the MCUs tested here and other devices, such as the LabJack U3. Their general functionality and the communication protocol between the host PC and the trigger device stays identical.

## Methods

**Fig. 1:**
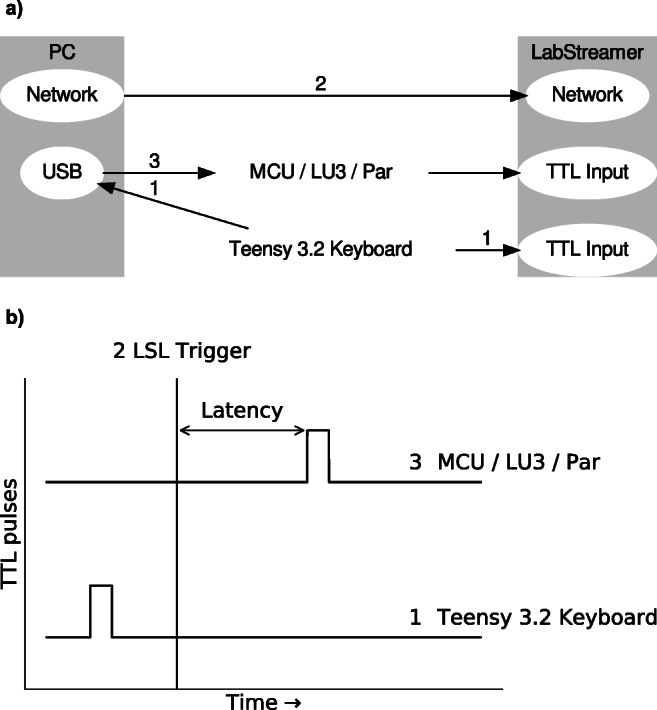
Testing setup. **a** Setup of the hardware. **b** Schematic representation of the signal time courses. For both panels, (1) the Teensy 3.2 keyboard simultaneously sends a key press to the PC and a TTL pulse to the LabStreamer; the PC receives the key press and sends (2) a timestamped LSL trigger via the network (marking timepoint zero), and (3) TTL pulse via the microcontroller (MCU), LabJack U3 (LU3), or parallel port (par) to the LabStreamer. This yields the latency of the tested device (**b**)

To compare the latency and jitter of MCUs with that of the parallel port, we devised a test setup using a LabStreamer device (NeuroBehavioral Systems; Albany, CA, USA), a standard Dell Optiplex 750 desktop computer with a native parallel port (the host computer), a Teensy 3.2 MCU to simulate keypresses, and a LabJack U3 (LabJack Cooperation; Lakewood, CA, USA) and four other MCUs to be tested against the parallel port (see Fig. 1). We established a communication protocol between the host computer and the LabStreamer device using the Lab Streaming Layer (LSL; https://github.com/sccn/labstreaminglayer). LSL is a software library for streaming timestamped measurement samples and TTL pulses over the local network. Clock offsets and uncertainty of the offset estimation between sender and receiver are periodically measured and subtracted out, so the receiver of a measurement sample (here: the LabStreamer) can record the time a TTL pulse was sent, rather than the time it was received.

The measurement process then proceeded as follows. The Teensy 3.2 MCU presented itself as a virtual keyboard to automatically send a keystroke to the host computer and simultaneously a TTL pulse to the LabStreamer (sampling the digital inputs at 10 kHz ) every 90 ms. Using LSL, the host computer and the LabStreamer then synchronized their clocks via a network connection. Upon receiving the simulated keypress signal, the host computer sent two additional signals: (i) a TTL pulse over the parallel port, LabJack U3, or a connected MCU, which was programmed to receive that signal via an emulated serial port running at 115200 baud per second, and (ii) an LSL trigger timestamped to the received keypress. The receiving MCU or LabJack U3 then read the signal byte and activated corresponding digital outputs, thus sending a TTL pulse to the LabStreamer (see Supplemental Material for the scripts). In each test run, at least 2500 measurements were made. Measurements with an LSL timestamp uncertainty below 0.01 ms, for example due to computational or network delays, were marked as invalid. Afterwards, we selected the first 2500 valid measurements from each test run for analysis.

We tested the LabJack U3 using two different methods of relaying the data: The standard “setFIOState” method, and the “writeRegister” method, which was recommended by the LabJack customer support to achieve faster latencies. Note that each method was tested on the same LabJack U3 device in separate measurement sessions.

Next to the parallel port and the LabJackU3, we tested four different, popular MCUs: (i) the widely used Arduino Uno as an example of an 8-bit MCU without native USB capabilities, (ii) the Arduino Leonardo (technically identical to the Arduino Pro Micro), an 8-bit MCU with an embedded native USB controller, (iii) the Teensy LC, an inexpensive 32-bit ARM MCU, and (iv) the Teensy 3.2, an affordable 32-bit MCU with enough communication interfaces and digital/analog in- and output ports for demanding experimental setups requiring the synchronization of several devices or processing of inputs, such as voice feedback. All devices (MCUs, parallel port, and LabJack U3) were tested with Python 3.6 and the Python bindings for the Psychtoolbox (Kleiner et al., 2007) on the Windows 7 and Ubuntu Linux 18.04 operating systems separately. To access the parallel port, the PsychoPy library was used (Peirce et al., 2019).

After data recording, we compared the device latencies of transmitting the byte from the PC to the labstreamer by calculating the mean, median, standard deviation, and interquartile range of the data for each device and operating system. Additionally, we computed these statistics averaged across devices within each operating system group to see whether the devices performed better on one or the other operating system. All computations and plotting were done in Python using the libraries Numpy (van der Walt et al., 2011), Pandas (McKinney, 2010), Matplotlib (Hunter, 2007), and Seaborn (Waskom et al., 2020).

## Results

**Fig. 2:**
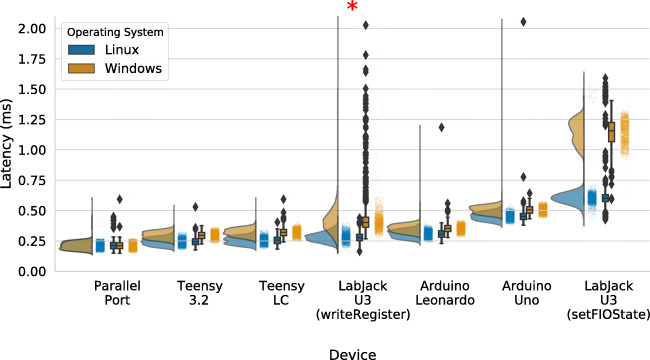
Raincloud plot (Allen et al., 2021) of TTL pulse latencies for the parallel port, all tested MCUs, and the LabJack U3. The LabJack U3 was tested using two different methods; (i) through the standard LabJack setFIOState method, and (ii) through writing data directly to the register with the writeRegister method. Under Windows, the LabJack U3 (writeRegister) had 11 more measurements between 2.3 and 7 ms that are not shown in the figure (mean = 4 ms), as indicated with the *red asterisk*

Mean and median latencies as well as standard deviation and interquartile ranges are reported in Table 1 and visually presented in Fig. 2. Across both operating systems, the parallel port had the lowest average latency and jitter. However, apart from the LabJack U3, all other tested devices consistently achieved latency and jitter comparable to that of the parallel port. For the LabJack U3, the mode of operation (writeRegister versus setFIOState) made a large difference. The writeRegister method resulted in smaller latencies, however this method also produced a large amount of outliers on Windows, but not on Linux (see Fig. 2). The setFIOState method, in contrast, resulted in the longest latencies of all tested devices, but did not produce as many outliers as the writeRegister method. Notably, the LabJack U3 operated with the setFIOState method was much faster under Linux compared to Windows. Averaging over devices (excluding the LabJack U3 as an outlier) within each operating system group showed a small performance benefit of using Linux over Windows. Devices operated on Linux were 0.042 ms faster (mean). The device latency between operating systems was nearly identical for the parallel port (mean latency Linux = 0.212 ms, mean latency Windows = 0.210 ms). Looking at the remaining devices (again, excluding the LabJack U3 as an outlier), the smallest effect of operating system on device latency was observed for the Arduino Leonardo (mean latency Linux = 0.308 ms; mean latency Windows = 0.352 ms), and the largest effect for the Teensy LC (mean latency Linux = 0.254 ms; mean latency Windows = 0.319 ms).

**Table 1:** Latencies for all tested devices in milliseconds. The table includes an entry for the Teensy 3.2 device that simulated a keyboard in the test setup. Its latency is negative because the simulated keystroke happened prior to the timepoint zero (the LSL Trigger, cf. Fig. 1)

Operating system	Device	Mean	SD	Median	IQR
Linux	Teensy 3.2 Keyboard	– 1.650	0.410	– 1.672	0.574
Linux	Parallel Port	0.212	0.032	0.211	0.05
Linux	Teensy 3.2	0.244	0.032	0.243	0.049
Linux	Teensy LC	0.254	0.032	0.255	0.052
Linux	LabJack U3 (writeRegister)	0.280	0.039	0.279	0.054
Linux	Arduino Leonardo	0.308	0.036	0.308	0.052
Linux	Arduino Uno	0.453	0.045	0.452	0.05
Linux	LabJack U3 (setFIOStatus)	0.616	0.120	0.603	0.057
Windows	Teensy 3.2 Keyboard	– 1.756	0.334	– 1.751	0.503
Windows	Parallel Port	0.210	0.030	0.209	0.049
Windows	Teensy 3.2	0.296	0.030	0.296	0.05
Windows	Teensy LC	0.319	0.033	0.319	0.05
Windows	Arduino Leonardo	0.352	0.032	0.353	0.05
Windows	LabJack U3 (writeRegister)	0.439	0.287	0.401	0.082
Windows	Arduino Uno	0.505	0.031	0.507	0.051
Windows	LabJack U3 (setFIOStatus)	1.144	0.099	1.157	0.158

*SD* standard deviation, *IQR* interquartile range

## Discussion

We evaluated four popular microcontrollers and a LabJack U3 device as potential replacements for the parallel port. All microcontrollers achieved latencies comparable to the parallel port in terms of both absolute latencies and jitter. The 8-bit MCUs (Arduino Uno, Arduino Leonardo) were generally slower than the 32-bit MCUs (Teensy LC, Teensy 3.2) but still consistently achieved latencies below 1 ms and below half a millisecond compared to the parallel port so even high-speed data recordings with sampling rates in the lower kilohertz range will therefore record triggers with a maximum delay and jitter of a few samples relative to a native parallel port. The LabJack U3 performed worse than all tested MCUs when accessed with code from the programming interface documentation (the setFIOState method). Direct access to the device’s internal memory through the writeRegister method improved the latency, at the cost of increased complexity for the user. It is important to note, however, that when operated under Windows, the writeRegister method resulted in a number of large outliers of up to 7 ms. This problem did not occur when operating the LabJack U3 with the writeRegister method under Linux, or when operating it using the setFIOState method under either operating system. Apart from these eye-catching outliers in that particular case, the remaining devices did not produce many outliers. Although there were a few outliers larger than half a millisecond in two tests (Arduino Leonardo and Arduino Uno on Linux), they were well below the usual unavoidable timing differences, such as due to missed screen refreshes and the latency of the input devices. Our testing setup also allowed us to measure the input lag due to the operating system without the underlying delays in real keyboards (see latency of Teensy 3.2 Keyboard in Table 1). This measured input lag provides a lower bound of unavoidable timing differences, that is, even if input (e.g., a keyboard or response box) and output (e.g., an event-trigger box based on an MCU) are hypothetically perfect and without delay, researchers still have to expect around 1.7 ms of delay with some jitter (see Table 1). This means that for example, in experiments marking a subject’s response in a recorded electroencephalogram (EEG) by sending an event trigger after receiving a keypress, the delay due to the operating system will be at least an order of magnitude above MCU TTL pulse latency (for a similar point, see Ulrich & Giray, 1989). Taken together, the results show that using MCUs (and with the mentioned caveats, the LabJack U3) to send digital event signals can reliably achieve adequate performance. Due to a lack of an Apple device with native parallel port support, we did not perform the tests under the macOS operating system. We speculate that the performance would have been slightly worse (longer latencies and increased jitter) compared to Windows and Linux, based on other results comparing the three operating systems (Bridges et al., 2020; Simpson, 2019). Yet for some macOS users, MCUs may still be an attractive option given the lack of alternatives.

Our findings build trust in the commercial products that have been introduced to the market in recent years to fill the gap left by parallel ports. However, the simplicity of our experimental setup and the devices we built for testing indicates that it may not be necessary to buy a commercial product: With some time and dedication, a replacement for the parallel port can be easily built (see Supplementary Materials for instructions). Such do-it-yourself (DIY) devices afford much greater flexibility than many commercially available devices: By choosing the MCU and other materials, as well as the software to run on the device, the end users themselves determine on which system the device will run. For the MCU devices presented in this study, we supply firmware as well as high-level Python code under a permissive license in the Supplementary Material. The LabJack U3 on the other hand is shipped with a proprietary firmware and a dedicated software interface provided by the LabJack Cooperation. With the growing literature on open-source hardware in the spirit of open science (White et al., 2019), many other building instructions and software examples are available on the Internet. Unlike many commercial products, MCUs and the additional components needed to build a replacement for the parallel port are relatively cheap (around €30 in total) and are thus also available to researchers and users whose budgets do not allow them to buy expensive new hardware (Chagas, 2018). It is still important to stress that each laboratory setup must be appropriately tested and benchmarked before data recording (Plant et al., 2004; Plant & Quinlan, 2013; Plant & Turner, 2009). However, the same applies to commercial hardware and software. In summary, considering the affordable prices, ease of use, performance, and flexibility of MCUs with a native USB controller, there are many reasons for deploying them in laboratory studies.

## Data Availability

The datasets generated and analyzed during the current study are archived and permanently available in the Zenodo repository under: 10.5281/zenodo.3838621. The supplemental material including the analysis code and code used for data recording is available as a website: https://sappelhoff.github.io/usb-to-ttl. The source of the website is archived on Zenodo and permanently available under: 10.5281/zenodo.3838692.
